# Artificial intelligence conversational agents in mental health: Patients see potential, but prefer humans in the loop

**DOI:** 10.3389/fpsyt.2024.1505024

**Published:** 2025-01-31

**Authors:** Hyein S. Lee, Colton Wright, Julia Ferranto, Jessica Buttimer, Clare E. Palmer, Andrew Welchman, Kathleen M. Mazor, Kimberly A. Fisher, David Smelson, Laurel O’Connor, Nisha Fahey, Apurv Soni

**Affiliations:** ^1^ Program in Digital Medicine, Department of Medicine, University of Massachusetts Chan Medical School, Worcester, MA, United States; ^2^ Department of Population and Quantitative Health Sciences, University of Massachusetts Chan Medical School, Worcester, MA, United States; ^3^ Ieso Digital Health, Cambridge, United Kingdom; ^4^ Division of Health System Science, Department of Medicine, University of Massachusetts Chan Medical School, Worcester, MA, United States; ^5^ Department of Emergency Medicine, University of Massachusetts Chan Medical School, Worcester, MA, United States; ^6^ Department of Pediatrics, University of Massachusetts Chan Medical School, Worcester, MA, United States

**Keywords:** artificial intelligence, chatbots, conversational agents, patient perspectives, qualitative, mental health, anxiety, cognitive behavioral therapy

## Abstract

**Background:**

Digital mental health interventions, such as artificial intelligence (AI) conversational agents, hold promise for improving access to care by innovating therapy and supporting delivery. However, little research exists on patient perspectives regarding AI conversational agents, which is crucial for their successful implementation. This study aimed to fill the gap by exploring patients’ perceptions and acceptability of AI conversational agents in mental healthcare.

**Methods:**

Adults with self-reported mild to moderate anxiety were recruited from the UMass Memorial Health system. Participants engaged in semi-structured interviews to discuss their experiences, perceptions, and acceptability of AI conversational agents in mental healthcare. Anxiety levels were assessed using the Generalized Anxiety Disorder scale. Data were collected from December 2022 to February 2023, and three researchers conducted rapid qualitative analysis to identify and synthesize themes.

**Results:**

The sample included 29 adults (ages 19-66), predominantly under age 35, non-Hispanic, White, and female. Participants reported a range of positive and negative experiences with AI conversational agents. Most held positive attitudes towards AI conversational agents, appreciating their utility and potential to increase access to care, yet some also expressed cautious optimism. About half endorsed negative opinions, citing AI’s lack of empathy, technical limitations in addressing complex mental health situations, and data privacy concerns. Most participants desired some human involvement in AI-driven therapy and expressed concern about the risk of AI conversational agents being seen as replacements for therapy. A subgroup preferred AI conversational agents for administrative tasks rather than care provision.

**Conclusions:**

AI conversational agents were perceived as useful and beneficial for increasing access to care, but concerns about AI’s empathy, capabilities, safety, and human involvement in mental healthcare were prevalent. Future implementation and integration of AI conversational agents should consider patient perspectives to enhance their acceptability and effectiveness.

## 1 Introduction

Mental illness affects over 57.8 million adults in the United States, accounting for more than 1 in 5 individuals (1). Despite the significant prevalence, many do not receive adequate care. Prior to the COVID-19 pandemic, only 41% of US adults diagnosed with anxiety, mood, or substance use disorders reported receiving treatment in the previous year (2–4). This treatment gap is largely attributed to a shortage of mental healthcare professionals, a persistent issue in the US healthcare system (5–8). Currently, more than 165 million people live in mental healthcare professional shortage areas in the US, with only 27.2% of mental health needs across all counties met by available psychiatrists (9). Untreated mental health can lead to worsening symptoms, decreased quality of life, and higher risks of comorbid conditions.

The growing popularity of Artificial Intelligence (AI) and Machine Learning offers promising digital medicine solutions for mental health access (10, 11). However, there remain significant uncertainties regarding patient acceptability (12). While AI has been successfully implemented in various healthcare domains, its application in mental health care is still emerging and understudied (13). Previous studies have demonstrated mixed results concerning the efficacy of AI conversational agents in delivering therapeutic interventions, with some research indicating benefits in accessibility and patient engagement (14–17), while others highlight concerns about the lack of empathy, accuracy, and the ability to handle complex mental health issues (18–21). Moreover, there is a notable gap in understanding patients’ perspectives on the acceptability of AI conversational agents, particularly among those with anxiety disorders. Existing research primarily focuses on the technical capabilities and preliminary outcomes of AI applications, often neglecting the critical aspect of patient experiences and perceptions (22, 23). This gap is particularly concerning given the increasing integration of AI in mental health services. Therefore, it is essential to explore patient viewpoints to ensure that these digital tools are not only effective but also acceptable and trusted by the users they aim to serve.

To address these knowledge gaps, we conducted a qualitative study using semi-structured interviews with 29 adults with self-reported mild to moderate anxiety. Participants were recruited from the UMass Memorial Health system and engaged in discussions about their experiences and perceptions of AI conversational agents in mental health care. Through this approach, we aimed to capture a diverse range of patient experiences, perceptions, and perspectives on the acceptability of using AI conversational agents for mental health support. Our analysis focused on identifying key themes and sub-themes that reflect the nuanced views of patients regarding AI-driven mental health interventions.

## 2 Methods

Our study utilized data collected through semi-structured qualitative interviews conducted with adult patients from the UMass Memorial Health system. The data collection period spanned from December 2022 to February 2023. These interviews were designed to explore patient experiences and perceptions regarding the use of AI conversational agents in mental healthcare. This study was approved by the UMass Chan Medical School Institutional Review Board (Protocol #1340270).

### 2.1 Participant eligibility and recruitment

The study included 29 adult participants with self-reported experiences of mild to moderate anxiety. Eligibility criteria required participants to be at least 18 years old, have a self-reported diagnosis of anxiety, be able to read, write, and speak English, and have the capacity to provide informed consent. Exclusion criteria included visual impairment without access to assistive technology, a history of suicide attempts or psychosis, recent changes in psychotropic medication, acute psychosis, or posing a danger to self or others.

**Figure 1:**
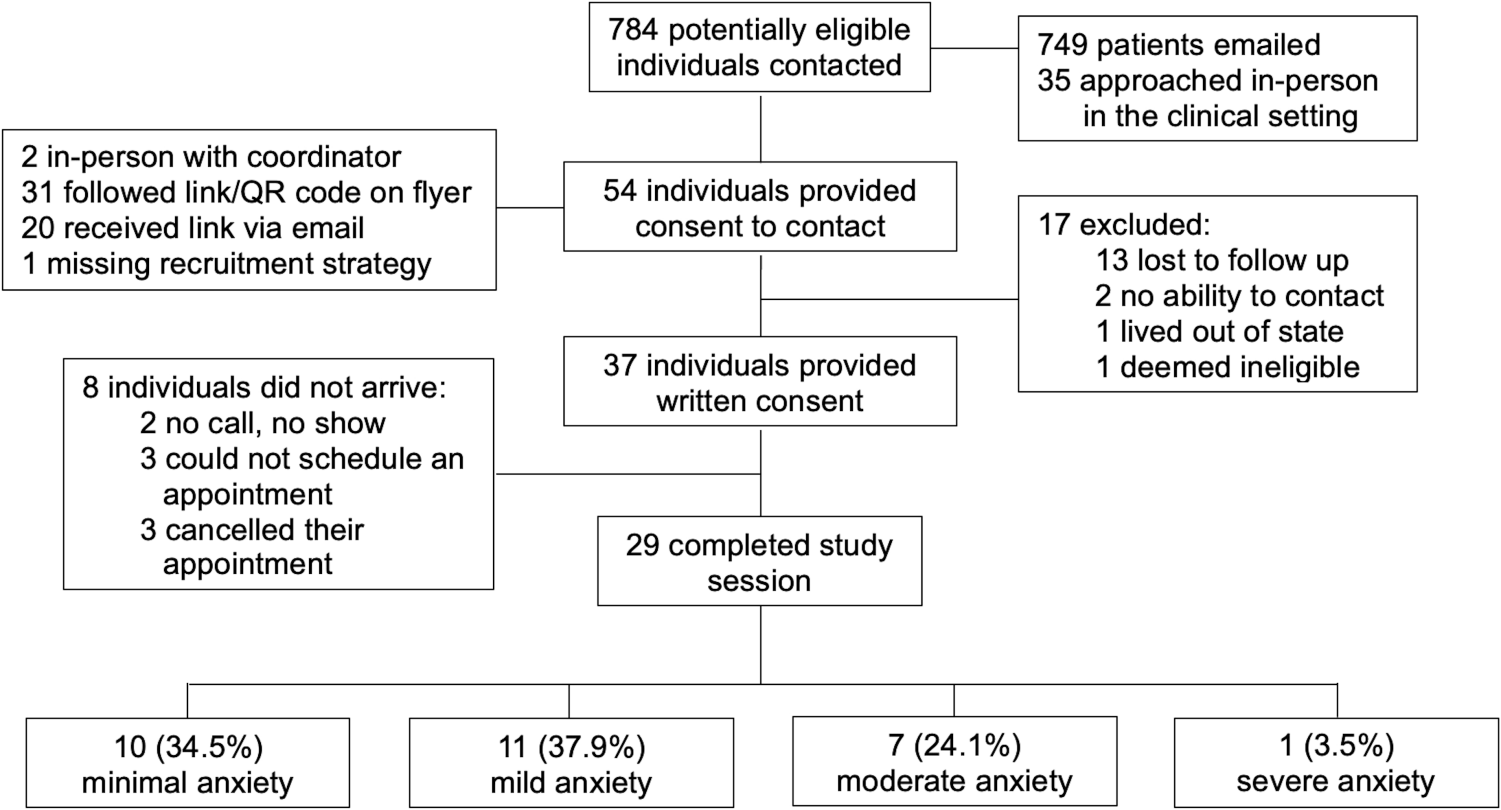
Participant consort diagram.

A HIPAA waiver was obtained to perform automated electronic medical record review and identify potentially eligible patients who received care at the UMass Memorial Health system. Eligible patients were contacted via recruitment emails, with additional recruitment through primary care clinicians and study flyers placed in clinic waiting rooms. Exclusion criteria were assessed both through chart review and initial screening phone interviews. Out of 784 potentially eligible individuals, 54 provided consent to contact, 37 provided written consent, and 29 completed the study procedures (**Figure 1**). Participants were compensated for their participation.

### 2.2 Procedure

Participants attended 90-minute study sessions, during which they completed demographic and anxiety symptom surveys and participated in a qualitative interview. Demographic data included age, gender, race, ethnicity, employment status, and education level. Anxiety levels were assessed using the Generalized Anxiety Disorder scale (GAD-7), a validated 7-item questionnaire with a 4-point Likert scale to measure anxiety severity over the past two weeks (24). Sum scores of 0-4 indicated minimal anxiety, 5-9 mild anxiety, 10-14 moderate anxiety, and 15-21 severe anxiety. The qualitative interviews were conducted remotely via Zoom by a user experience researcher and were supported by an in-person study coordinator. Interviews were recorded, transcribed, and observational notes during each session were taken. The interviews had three sequential components: 1) participants were asked questions about their past experiences with AI conversational agents and perceptions of AI conversational agents in mental healthcare, 2) participants engaged with a mental health conversational agent app to ground and standardize their experiences, and 3) participants were asked additional general questions related to the acceptability of AI conversational agents in mental healthcare, and more. This manuscript is comprised of data collected only from the first and third parts of the interview. The second part of the interview was comprised of text-based conversation with a prototype AI conversational agent using a tree-based dialogue system underpinned by natural language understanding. The prototype agent did not respond to user input with any generative responses and performance of the prototype is not the focus of this manuscript.

### 2.3 Data analysis

Data were analyzed using rapid qualitative analysis as informed by Hamilton (25, 26). A rapid qualitative analysis approach was used considering the resources available for this pilot study, and because it yields rigorous, structured, and actionable data in a shorter timeframe compared to traditional qualitative analysis methods (27–29). The benefit of rapid qualitative analysis is its ability to quickly identify insights (e.g., gaps in care, facilitators/barriers, etc.) and guide decision-making and implementation strategies for targeted healthcare issues (30–33). First, a summary template was created so that a domain was mapped onto each interview question. Second, the transcript data and observational notes for each participant was divided up by three researchers, summarized into each domain, and organized into a matrix. Third, the three researchers reviewed one another’s summaries so that each transcript was reviewed twice by a researcher (and at least once by a researcher who was not present at the interview), and summaries were combined. The three researchers met regularly with two senior researchers experienced in qualitative research to discuss similarities and differences in domain summaries, reduce duplication, synthesize themes and sub-themes within and across domains, and identify important quotes. Discrepancies across researchers were discussed and reconciled to reach consensus in data interpretation over regular team meetings. By combining independent analysis, triangulation through cross-reviewing with multiple researchers, and iterative discussion of thematic interpretations, we sought to reduce bias and promote rigor in our analysis. In this study, only data pertaining to past experiences, perceptions, and acceptability of digital mental health tools in mental healthcare were analyzed.

## 3 Results

### 3.1 Participant characteristics

The sample consisted of 29 adults with self-reported diagnosis of anxiety. The demographic characteristics of the participants are detailed in **Table 1**. Participants tended to be younger, with 34.5% aged 18-24, 31% aged 25-34, and 34.5% aged 35 and older. The majority of participants were non-Hispanic White (65.5%) and female (72.4%). Most participants were employed (65.5%) or in school (27.6%). Educational attainment varied, with 44.8% holding a high school diploma or some college education, 34.5% holding a bachelor’s degree, and 20.7% holding a master’s degree or higher. Anxiety levels were assessed using the GAD-7 scale, with 34.5% of participants screening for minimal anxiety, 37.9% for mild anxiety, 24.1% for moderate anxiety, and 3.4% for severe anxiety symptoms.

**Table 1:** Study participant characteristics (n=29).

Demographics	n (%)	GAD7			
		Minimal n= 10	Mild n= 11	Moderate n= 7	Severe n= 1
Age					
18-24	10 (34.5%)	0	5 (50%)	4 (40%)	1 (10%)
25-34	9 (31%)	5 (55.6%)	4 (44.4%)	0	0
35-44	5 (17.2%)	4 (80%)	0	1 (20%)	0
45-54	1 (3.5%)	0	1 (100%)	0	0
55-64	2 (6.9%)	0	1 (50%)	1 (50%)	0
65 and over	2 (6.9%)	1 (50%)	0	1 (50%)	0
Gender					
Male	6 (20.7%)	4 (66.7%)	1 (16.7%)	1 (16.7%)	0
Female	21 (72.4%)	6 (28.6%)	9 (42.9%)	5 (23.8%)	1 (4.8%)
Non-binary	0 (0%)	0	0	0	0
Transgender	1 (3.5%)	0	0	1 (100%)	0
Prefer not to answer	1 (3.5%)	0	1 (100%)	0	0
Race					
American Indian or Alaska Native	0 (0%)	0	0	0	0
Asian	5 (17.2%)	2 (40%)	2 (40%)	1 (20%)	0
Black or African American	4 (13.8%)	0	3 (75%)	1 (25%)	0
Native Hawaiian or Pacific Islander	0 (0%)	0	0	0	0
White	19 (65.5%)	8 (42.1%)	6 (31.6%)	5 (26.3%)	0
Prefer not to answer	1 (3.5%)	0	0	0	1 (100%)
Hispanic, Latino or Spanish origin or ancestry					
No	25 (86.2%)	9 (36%)	10 (40%)	6 (24%)	0
Other or mixed Hispanic, Latino or Spanish origin	3 (10.3%)	1 (33.3%)	1 (33.3%)	0	1 (33.3%)
Missing	1 (3.5%)	0	0	1 (100%)	0
Employment status					
Employed	19 (65.5%)	9 (47.4%)	5 (26.3%)	4 (21.1%)	1 (5.3%)
Not working and not actively seeking work	1 (3.5%)	0	0	1 (100%)	0
Student	8 (27.6%)	1 (12.5%)	5 (62.5%)	2 (25%)	0
Prefer not to answer	1 (3.5%)	0	1 (100%)	0	0
Highest level of education					
High School Graduate/Some College No Degree	13 (44.8%)	3 (23.1%)	6 (46.2%)	4 (30.8%)	0
Bachelor’s degree (e.g. BS, BA, AB, BBA)	10 (34.5%)	2 (20%)	5 (50%)	2 (20%)	1 (10%)
Master’s Degree or higher (e.g. MS, MBA, PhD)	6 (20.7%)	5 (83.3%)	0	1 (16.7%)	0

### 3.2 Past experiences with AI conversational agents

Participants had diverse past experiences with AI conversational agents, primarily in retail and customer service contexts (**Table 2**). Positive experiences were often associated with newer technologies like ChatGPT, which impressed participants with its conversational capabilities. However, these positive experiences were tempered by an awareness of AI’s current limitations. Many participants cited negative experiences, including frustrations with conversational agents’ lack of personalization and empathy, and their inability to understand specific requests. As one participant noted, “Most of my experience with using chatbots has been kind of irritating … It always seems to be when you’re having a customer service problem or you need help with your bank account or this or that and all you ever want is to talk to a real person and you feel like you have to go through 400 chatbots before you can get an answer” (P24: Female, 28, Minimal Anxiety).

**Table 2:** Past experiences with AI conversational agents.

Themes & Sub-Themes	Representative Quotes
Positive Experiences	“Actually, I’m pretty impressed by what is it called – ChatGPT or something like that – that just recently came out. My son showed me an essay on a topic that he just put into the screen, and it came up with an essay within a minute kind of thing. I was really impressed by that, so I think that artificial intelligence is only as good as the foundation upon which it was built – that information upon which it was built. So, I think in general I think it’s a beginning. It’s in its beginning stages and probably will evolve to be something pretty great and useful.” (P14: Female, 65, Moderate Anxiety) “I feel comfortable with it. I mean, I think that like, I’m in my late twenties, so this is something that I grew up with. I haven’t seen too much. Like most of the chatbot experience I have at this point is through like stores or online retailers where the experience varies widely. Sometimes it’s really good and you get the answer you’re looking for or you’re connected directly to a live person who is able to answer questions. But other times it’s not as great of an experience where it might be difficult to be connected to someone or for them to actually get a sense of what you’re trying to ask and what the answer is.” (P13: Male, 29, Minimal Anxiety)
Negative Experiences	“Most of my experience with using chatbots has been kind of irritating … It always seems to be when you’re having a customer service problem or you need help with your bank account or this or that and all you ever want is to talk to a real person and you feel like you have to go through 400 chatbots before you can get an answer” (P24: Female, 28, Minimal Anxiety) “I feel like often times when I use a chatbot it just sends me links to things that I have already like looked at rather than answering the question that I actually have.” (P12: Female, 31, Minimal Anxiety) “I think I’m a little bit like on the edge about it, because … I’ve never really seen anything like that before and my experience using chatbots hasn’t really been positive because it’s like it’s not really a real person who’s able to, you know, feel or anything.” (P18: Female, 19, Moderate Anxiety) “My experience with online chatbots has been pretty bad. It’s like they don’t understand what I’m talking about. So basically, I’m talking to a computer. I understand it’s a very sophisticated computer, but nevertheless in my experience it doesn’t know what I’m really getting at.” (P17: Male, 66, Minimal Anxiety)
Lack of Experience	“I don’t know much about it. I’m not very good with technology, you know. But it doesn’t scare me at all, or anything. I’m interested to see how this goes, because I’ve heard that it’s being used more and more and I’m wondering how realistic it is.” (P03: Female, 21, Mild Anxiety)

### 3.3 Perceptions of AI mental health conversational agents

When asked about participants expectations about an app that “addresses mental health concerns via a conversation delivered by an AI powered agent,” participants expressed a range of perceptions as reflected in **Table 3**.

**Table 3:** Positive and negative perceptions of AI conversational agents in mental healthcare.

Themes & Sub-Themes	Representative Quotes
Positive Perceptions	
Increased accessibility	“I think if it can be helpful, it’s a great additional tool. You know mental healthcare is pretty limited and not available to most people so I think if it can be used to sort of make mental health care available with integrity to more people, I think that’s a great idea” (P14: Female, 65, Moderate Anxiety). “I think it’s good that it can increase accessibility. I think it’s tough, because having one approach for the dissemination of information can be hard for people to grasp, especially when you’re stuck in a certain thought process or a way of thinking. But I think especially now that we have such a surge in the need for mental healthcare (and it’s really, really hard to access for patients), I think it’s a good idea to have at least something there that can kind of like address that gap.” (P26: Female, 23, Mild Anxiety) “It’s funny because I work for an AI company, not in mental health … and I like the direction that AI goes is going in. I think there’s a lot of opportunity with it. I have experience using … not an AI driven app, but where it fell short for me (the reason why I ended that) was because I didn’t feel the connection with the therapist because it was all via chat and it was not real time, so there was a lot of delays in getting a response and everything. So I would hope that the AI approach would be more real time.” (P25: Female, 35, Minimal Anxiety)
Cautious optimism	“I feel I’m kind of like ‘wishy washy’ with it, but I feel like it could be used for this type of work, like this field. Because obviously we can’t have therapists available for everyone at every second of every day, so I could see how using AI would be beneficial to something like this.” (P09: Transgender, 22, Moderate Anxiety) “I’m interested by it, because it’s like obviously so different, because it’s a computer, so what does the computer know about mental health, but … I’m intrigued.” (P06: Female, 24, Severe Anxiety) “It seems like sort of a sensitive topic but something that I think is worth exploring. But I don’t know if I would inherently trust it without experiencing it first.” (P21: Male, 35, Moderate Anxiety)
Negative Perceptions	
Lack of empathy	“Not the best. I do feel that there needs to be some sort of human connection, or like intellect there because I don’t think AI will always get what somebody is feeling.” (P19: Female, 19, Mild Anxiety) “I’m on the fence about it. I think it’s difficult for a lot of people … I mean checking into work, they just got all these kiosks and I find it that people are very frustrated with it, especially the elderly. I think sometimes it’s the hospital setting that already gives them anxiety, and then they see this machine or not a live person. It adds to it. It sounds like it’s all artificial. I don’t think I would like that … I don’t know if I’d always want to talk to somebody that was artificial. [It’s] impersonal and mental health is a personal thing.” (P22: Female, 56, Moderate Anxiety) “I’m not sure it could offer like empathy like a person.” (P12: Female, 31, Minimal Anxiety)
Technical limitations	“Just concerned that the digital therapy might not understand what I’m totally feeling or may not respond in the way that I want them to respond” (P29: Female, 21, Moderate Anxiety). “I feel like they can be a little unreliable, especially if you use language that it doesn’t understand or it perceives as something different.” (P19: Female, 19, Mild Anxiety) “I guess I’m just a little skeptical because a lot of thinking and assessment is just done by like a human mind and it takes a lot of training and knowledge to be able to look into someone’s mental state and mind. So I’m skeptical that an AI can do that out of a text message” (P11: Female, 26, Minimal Anxiety). “It’s a double-edged sword for immediate things. Like when it’s being used for mental health, it has to know – like there’s so many scripts running in the background as you’re talking – the AI has to know … [when] to contact the emergency personnel right now, people at the ready, especially if someone is speaking about suicide.” (P15: Male, 35, Minimal Anxiety)
Data privacy	“I mean, there’s always the chance that the system could get hacked or something … I feel like if you were having super intense like anxious moments, or depression moments, that if it got leaked you wouldn’t feel good about that being leaked. If that makes sense.” (P03: Female, 21, Mild Anxiety) “I think trust would be a big thing. Yeah, it’s asking people to tell me the details of your life. But where does this go, who is monitoring this? I think those are questions that people would have.” (P10: Female, 27, Minimal Anxiety) “Not great … I would not feel comfortable giving out any personal information or like in-depth information about what I’m seeking treatment for via AI. Because I think AI works by using like a database of what everyone before it has said – so I think it runs the risk of stereotyping or grouping people into diagnoses via AI, and not via a licensed professional.” (P27: Prefer Not to Answer, 26, Mild Anxiety)

#### 3.3.1 Positive perceptions

A majority (n=21) reported positive but hesitant opinions, recognizing AI’s potential to increase care accessibility and efficiency, yet remaining skeptical about its current capabilities (**Table 3**). Participants noted the potential for increasing accessibility especially for those who find it challenging to access traditional therapy. One participant remarked, “I think if it can be helpful, it’s a great additional tool. You know mental health care is pretty limited and not available to most people, so I think if it can be used to sort of make mental health care available with integrity to more people, I think that’s a great idea” (P14: Female, 65, Moderate Anxiety). Others appreciated the convenience and immediate availability of AI conversational agents, which could serve as a valuable supplement to traditional therapy.

#### 3.3.2 Negative perceptions

Around half of participants (n=17) conveyed skepticism, doubt, or concerns about the capabilities and application of AI mental health conversational agents (**Table 3**).

##### 3.3.2.1 Lack of empathy

Many participants doubted AI’s ability to provide empathetic and thoughtful responses, a critical component of effective mental health care. A participant expressed skepticism, stating, “I do feel that there needs to be some sort of human connection, or like intellect there because I don’t think AI will always get what somebody is feeling.” (P19: Female, 19, Mild Anxiety). A few individuals also emphasized that AI’s perceived lack of empathy may create higher barriers for older individuals to use AI conversational agents, especially when compounded with their general unfamiliarity with this emerging technology.

##### 3.3.2.2 Technical limitations

Issues such as the conversational agents’ inability to understand complex mental health needs and generate appropriate responses were significant concerns. One participant shared, “Just concerned that the digital therapy might not understand what I’m totally feeling or may not respond in the way that I want them to respond” (P29: Female, 21, Moderate Anxiety). In this context, several were also skeptical of AI’s therapeutic potential for more severe mental health conditions and questioned AI’s ability to adequately navigate and address emergency situations, such as suicidal crises.

##### 3.3.2.3 Data privacy concerns

Participants were worried about the security and confidentiality of their personal data. Fears of hacking were elevated due to the sensitive nature of data related to mental illness and vulnerable emotional states. For instance, one participant noted, “I mean there’s always the chance that the system could get hacked or something … I feel like if you were having super intense like anxious moments or depression moments that if it got leaked you wouldn’t feel good about that being leaked. If that makes sense” (P03: Female, 21, Mild Anxiety). Some participants associated data privacy with trust and specified that they were wary of their data being sold, shared, or monitored. One participant highlighted concerns with providing in-depth personal information due to potential for AI model training leading to bias and stereotyping if an AI conversational agent were to diagnose mental illnesses.

### 3.4 Acceptability of AI conversational agents in mental healthcare landscape

When asked “how do you feel about the use of Artificial Intelligence in mental healthcare?” participants discussed the acceptability of AI conversational agents within the mental healthcare landscape, as reflected in **Table 4**. In this section, only 17 out of 29 participants were asked questions around acceptability of AI conversational agent apps due to time constraints in the interview.

**Table 4:** Acceptability of AI conversational agent applications in mental healthcare.

Themes & Sub-Themes	Representative Quotes
Acceptable Amount of Human Involvement	
Some amount of human involvement necessary	“I think [accessing digital therapy in an app] is great as long as it’s like in conjunction with actual therapy. I don’t think it’s good to have it on its own. I think it needs to be in contrast with in-person” (P05: Female, 25, Mild Anxiety). “I’m still on the fence about that. I guess if somebody is reluctant to share information about their issues with the medical professional, this might be the right vehicle for them. But I don’t think it can be used solely for direction and guidance. I think that live people need to be supplemented with the app. I think the human involvement should be the first involvement, and then they suggest the app to be used in conjunction with either therapy, medication, or a little of both.” (P23: Female, 59, Minimal Anxiety).
Concern with replacement of human therapists	“I have concerns with] people thinking that it’s the only type of mental health support that they need like whether they’re going to actual therapy or they should be going to actual therapy and they’re using this instead. Or using this as something that might help them get off any type of medication that they’re using. Or if they’re feeling all right, they might feel a lot more confident to go off of any medication that they’re using.” (P19: Female, 19, Mild Anxiety).
No human involvement necessary	“I feel good about it, because when you’re in person and discuss what are you feeling, sometimes you might not say how you feel because you feel like ‘oh, maybe that person is judging.’ But this is more like you’re writing, and you don’t have a person there so this will make it more easier to share how you feel.” (P07: Female, 26, Mild Anxiety) “I feel like [digital therapy with no human involvement] would also still be like beneficial to the patient because of course, it’s better than nothing … and where it’s so hard for a lot of people to find therapists that accept their insurance that are in the area … it would make it a lot easier for people – like it’d be a lot more accessible for people that would traditionally not have that option.” (P09: Transgender, 22, Moderate Anxiety)
More Acceptable Functions for AI Conversational Agents	“I can’t imagine it replacing a therapist. But I think in the more administrative tasks it could work … like initial screenings, and like I said, matching the patient to the correct health care provider.” (P24: Female, 28, Minimal Anxiety) “I think it can be really helpful in some cases including in the healthcare setting and research. I think things that are subjective like pathology or radiology reports – things that can be misread – AI can take the subjective out and make more objective assessments.” (P11: Female, 26, Minimal Anxiety) “I think it’d be okay if I was just renewing prescriptions or didn’t necessarily need to talk to the doctor and it was just a follow-up or something. But I don’t know if I’d always want to talk to somebody that was artificial.” (P22: Female, 56, Moderate Anxiety) “I think, for baseline interventions it can play a role in mental health, but I think for deeper dives it may be more difficult, because it’s AI, and I think it’s hard to really have an AI delve into feelings.” (P23: Female, 59, Mild Anxiety)

#### 3.4.1 Acceptable amount of human involvement in AI-driven therapy

A majority of participants (n=11) felt that implementing AI conversational agents without any human involvement was not acceptable and believed AI conversational agents should not replace therapy with mental health professionals.

##### 3.4.1.1 Some amount of human involvement necessary

Many participants expressed that AI conversational agents could still be helpful but preferred them in combination with therapy led by a person (either in-person or over telehealth) rather than as a stand-alone service. One participant noted, “I think [accessing digital therapy in an app] is great as long as it’s like in conjunction with actual therapy. I don’t think it’s good to have it on its own. I think it needs to be in contrast with in-person” (P05: Female, 25, Mild Anxiety). This theme is consistent across interviews, indicating a preference for AI conversational agents to act as supplementary tools rather than replacements for human therapists.

##### 3.4.1.2 Replacement of human therapists

Some participants were worried that AI conversational agents might be viewed as replacements for traditional therapy, which they believed could be dangerous. One participant mentioned, “[I have concerns with] people thinking that it’s the only type of mental health support that they need like whether they’re going to actual therapy or they should be going to actual therapy and they’re using this instead” (P19: Female, 19, Mild Anxiety).

##### 3.4.1.3 No human involvement necessary except for emergencies

A minority of participants felt that no human involvement in AI conversational agents was acceptable, emphasizing the benefits of anonymity in decreasing barriers to starting therapy due to stigma or fear of judgment. One participant mentioned, “I feel good about it, because when you’re in person and discuss what are you feeling, sometimes you might not say how you feel because you feel like ‘oh, maybe that person is judging’. But this is more like you’re writing, and you don’t have a person there so this will make it more easier to share how you feel.” (P07: Female, 26, Mild Anxiety). Participants also often contextualized their acceptance within the current reality of limited access to mental health resources. As an important distinction, almost all participants expected human intervention to be absolutely necessary in an AI conversational agent app if a patient were to mention self-harm, suicidal ideation, or ideas of harming others.

#### 3.4.2 More acceptable functions of AI conversational agents

Although many participants expressed hesitancy towards AI’s application for higher-order tasks without human involvement such as providing therapy or conversation, some felt that AI could be useful and more acceptable for more basic tasks. These tasks included renewing prescriptions, managing follow-up appointments, matching patients to healthcare providers, diagnosis support, administrative tasks (e.g., initial screenings), and baseline mental health interventions that fall short of delving into complex emotions. One participant mentioned, “I can’t imagine [AI tools] replacing a therapist. But I think in the more administrative tasks it could work … like initial screenings and, like I said, matching the patient to the correct health care provider.” (P24: Female, 28, Minimal Anxiety).

## 4 Discussion

This study explored the experiences, perceptions, and acceptability of AI conversational agents for mental health support among adults with self-report of mild to moderate anxiety. Our key findings revealed that while participants recognized the potential benefits of AI conversational agents in increasing accessibility to mental health care, they also expressed significant concerns regarding the potential lack of empathy, technical limitations, and data privacy. Most participants preferred AI conversational agents as supplementary tools rather than replacements for human therapists, emphasizing the need for some level of human involvement to ensure effective mental health care. These findings underscore the importance of addressing these concerns to enhance the acceptability and effectiveness of AI-driven mental health interventions.

The rapid diffusion of generative AI tools, demonstrated most poignantly by the rise of popularity and use of tools like ChatGPT, Claude, Gemini, etc. has positioned AI conversational agents as promising tools in supplementing care in mental health (34–36). Research has demonstrated that mental health conversational agents can increase engagement with therapeutic content and improve mental health symptoms (37–39). Many benefits of AI conversational agents identified in our study were similar to those found in previous studies, such as lowering barriers to care, improving access to therapeutic content, and alleviating the burden on current mental health professionals (14, 15, 17, 40). Multiple participants highlighted convenience and anonymity as key components of AI conversational agents that can lower barriers to care. These benefits may be even greater for those who may avoid seeking care due to stigma, fear of judgment, or anxiety interacting with real people. Previous studies have found that conversational agents can decrease stigma (41) and may increase the likelihood that patients disclose emotional and sensitive information compared to when interacting with other humans (42–44). The benefits of AI conversational agents may enhance equity of care for underserved groups, including rural, low-income, LGBTQ+, and racial/ethnic communities who already struggle to find affordable and culturally competent services (19, 45, 46).

Our analysis identified increased access to care as one of the main benefits of AI conversational agents. Participants often contextualized this benefit within the current mental health crisis where the need for care is substantially outpacing the supply of available mental health workers (47, 48). Despite the number of psychiatrists entering the workforce increasing by 26.3% from 2016 to 2021 (49), it is estimated that 6,129 additional psychiatrists are still needed as of 2024 to alleviate the current national shortage (47). The widespread adoption of smartphones (50) makes AI conversational agents a feasible and scalable solution to bridge this care gap without requiring patients to wait for or travel to appointments. Our study’s findings support the potential of AI conversational agents to increase access to care, a crucial benefit amid the current mental health crisis, and further support the role of AI conversational agents in assisting mental health professionals by providing data-driven insights and personalized, supportive interactions, thereby alleviating their workload.

Despite the recognized benefits, participants in our study raised significant concerns about the perceived lack of empathy, technical reliability, and data privacy of AI conversational agents. These issues are well-documented in the literature (14, 15). Gerke et al. highlighted the ethical and legal challenges of AI-driven healthcare, focusing on data security, safety, and algorithmic fairness, all of which are especially critical in mental health care (51). Patients are more likely to engage with digital health tools when they feel their data is secure (40, 52, 53). Establishing transparent data handling practices and ensuring user trust are essential for successful AI conversational agent adoption. Concerns about AI’s ability to understand and respond to complex mental health needs were also prevalent, especially in context of emergency mental health crises. In reference to these concerns, along with worries about the lack of empathy and personalization in AI responses, participants felt that AI conversational agents should be used as supplementary tools and retain some level human involvement. Consistent with previous research (40, 54), our findings highlight important implementation considerations around the acceptability of AI conversational agents as standalone mental health interventions. These gaps in current AI mental health applications emphasize the need for advanced algorithms to handle the nuances of mental health needs, improve empathy and personalization, and ensure data privacy (13). Addressing these technical and ethical concerns is crucial for enhancing the acceptability, efficacy, and adoption of AI conversational agents, building on previous research, and guiding future developments (51, 55). Our study provides a patient-centered perspective, highlighting real-world concerns and expectations. Additionally, it presents a roadmap for evolution of technologies in this space as they build on early successes of tree-based dialogue systems with more large-language model incorporated agents.

### 4.1 Limitations

While our study provides valuable insights into the perceptions and acceptability of AI conversational agents for mental health support among adults with mild to moderate anxiety, there are several limitations to consider.

First, the sample size comprised predominantly young, non-Hispanic White women, which may limit the generalizability of the findings. The limited diversity of our sample may have affected our results as men and younger people have been shown to be more open to AI technologies in healthcare (56, 57) and be more likely to have used AI conversational agents (58, 59), though these findings have been inconsistent across studies (60–63). This selection bias could mean that the experiences and perceptions of other demographic groups were not adequately represented. To mitigate this limitation, future studies should aim to include a more diverse sample size to enhance the comprehensiveness of the results. Similarly, the study’s setting in a single healthcare system may limit the applicability of the findings to other contexts. The specific characteristics of the UMass Memorial Health system and its patient population may not reflect those of other healthcare systems. Considering the growing concerns around potential biases of AI algorithms (64), lack of diversity in training datasets (65), and inequitable access to care (66), future studies with diverse groups of patients and across different healthcare settings and geographic locations​ are warranted.

Second, the rapidly evolving nature of AI technology means that the capabilities and limitations of AI conversational agents are continuously changing. The findings of this study are based on the state of AI technology from December 2022 to February 2023 and may not fully capture perceptions around technology advances since then. The timing of our study coincided with the rise of AI conversational agents use in the general public, most notably with the release of ChatGPT by OpenAI on November 30, 2022 (67). It is likely that perceptions of participants may be rapidly evolving as they experience or learn about new capabilities and acceptability of AI conversational agents. Previous studies have shown that prior knowledge of or familiarity with AI in healthcare can have positive moderating effects on perceptions of AI in healthcare (68–70). However, we did not formally assess participants’ knowledge of or familiarity with AI conversational agents or ChatGPT, which may have introduced unmeasurable bias and variability in participants’ perceptions of AI conversational agents. It is important for ongoing research to continuously evaluate and update the understanding of AI conversational agent applications in mental health care as the technology evolves.

Despite these limitations, our study provides a foundational understanding of patient perceptions and acceptability of AI conversational agents in mental health care. By recognizing and addressing these limitations, future research can build on our findings and contribute to the development of more effective and acceptable AI-driven mental health interventions.

### 4.2 Implications

The findings of our study have practical implications for patients, providers, payers, and policymakers in the healthcare ecosystem. AI conversational agents can bridge gaps in mental health care access, especially for medically underserved populations. By improving accessibility and reducing stigma, AI conversational agents provide a convenient, anonymous platform for individuals hesitant to seek traditional therapy, encouraging proactive mental health management. For providers, AI conversational agents can extend reach and efficiency by handling routine inquiries and initial support, allowing clinicians to focus on more complex cases. This approach can lead to better resource allocation and improved patient outcomes. Payers benefit from the cost-effectiveness of AI conversational agents, as they reduce the burden on mental health professionals and enable early intervention, lowering overall healthcare costs. Insurance companies should consider covering AI-driven mental health services to promote wider adoption. Policymakers play a critical role in regulating AI use in mental health care. Our study highlights the need for robust data privacy and security standards to protect patient information. Policymakers should develop regulations ensuring the ethical use of AI technologies, addressing data privacy, algorithmic bias, and transparency. Policies supporting research and development in AI mental health applications can drive innovation and improve efficacy and acceptability. The integration of AI conversational agents in mental health care has the potential to transform the landscape of mental health services. By addressing the limitations identified in our study and leveraging AI’s strengths, stakeholders can create a more accessible, efficient, and effective mental health care system. The future of mental health care depends on collaborative efforts of patients, providers, payers, and policymakers to harness AI’s power while ensuring ethical and patient-centered practices.

## Data Availability

The datasets presented in this article are not readily available because they consist of qualitative interviews. The data are not publicly available to protect the identities of participants. Further inquiries can be directed to the corresponding author. Requests to access the datasets should be directed to Apurv Soni, apurv.soni@umassmed.edu.
